# PREDICTING OLDER ADULTS’ RESIDENTIAL CARE RELOCATION RISKS BASED ON CAREGIVER NETWORK CHARACTERISTICS

**DOI:** 10.1093/geroni/igae098.1290

**Published:** 2024-12-31

**Authors:** Natasha Nemmers, Wenhua Lai, Srabani Haldar, Sophia Tsuker, Vicki Freedman, Amanda Leggett

**Affiliations:** Wayne State University, Detroit, Michigan, United States; Wayne State University, Detroit, Michigan, United States; Wayne State University, Detroit, Michigan, United States; Wayne State University, Detroit, Michigan, United States; University of Michigan, Ann Arbor, Michigan, United States; Wayne State University, Detroit, Michigan, United States

## Abstract

When older adults face increasing care needs or limited support, remaining safely and comfortably at home becomes challenging. Extant research has primarily concentrated on characteristics of the older adult or their primary caregiver on nursing home admission. This study examines the risk of older adults transitioning to residential care (e.g., assisted living, nursing home), focusing on the influence of their caregiver network or involvement of multiple caregivers. Using the National Health and Aging Trends Study, we conducted competing risk regressions following 7,085 community-dwelling older adults moving to residential care, accounting for death, across rounds 1-9 (2011 – 2019). We assessed network composition, size, shared tasks, and the number of in-network specialists or generalists, while controlling for individual sociodemographic and health factors. Individuals with caregiver networks that shared medical tasks had the highest risk of moving, followed by those sharing household tasks. Conversely, shared mobility or self-care and transportation responsibilities were associated with lower risks. Having more generalists increased the risk, while any number of specialists was non-significant. Larger networks were associated with heightened risk, though having close family like a spouse was protective. The findings underscore that caregiver network characteristics are critical to older adults’ ability to age in place. Specifically, older adults with larger caregiver networks, lacking a spouse or child, and providing complex care are at greater risk for relocating. Understanding caregiver networks can guide interventions related to care network coordination and resource allocation to help avoid or postpone a residential care move.

